# Do Faster-Growing Holoparasitic Plant Species Exhibit Broader Niches and Wider Global Distributions?

**DOI:** 10.3390/plants14060831

**Published:** 2025-03-07

**Authors:** Quanzhong Zhang, Jinming Hu

**Affiliations:** 1 Institute of International Rivers and Eco-Security, Yunnan University, Kunming 650500, China; zhangqz@mail.ynu.edu.cn; 2 Faculty of Geography, Yunnan Normal University, Kunming 650500, China

**Keywords:** *Cuscuta* subgenus *Grammica*, ecological niche breadth, growth rate, global distribution pattern, holoparasitic plants, parasitic organisms

## Abstract

Parasitic organisms, as an important component of ecosystems, have long been a focal point in ecological research, particularly concerning the relationship between their growth characteristics, ecological niche, and distribution patterns. This study selects the holoparasitic plant species *Cuscuta campestris* Yunck., *Cuscuta australis* R.Br., and *Cuscuta chinensis* Lam. from the *Cuscuta* subgenus *Grammica* as model species to explore the relationship between the growth rate, ecological niche breadth, and global distribution patterns of parasitic plants. Through greenhouse experiments and data analysis, the main findings of this study indicate a strong positive correlation between the growth rate, ecological niche breadth, number of global occurrence points, and global distribution area for *C. campestris*, *C. australis*, and *C. chinensis*. The significant correlation between growth rate and ecological niche breadth suggests that the intrinsic growth characteristics of parasitic plants may significantly influence their realized ecological niche. Furthermore, the experimental results show that when *C. campestris*, *C. australis*, and *C. chinensis* parasitize non-native hosts from the Americas, they produce greater biomass than when parasitizing native hosts from China. In conclusion, this study provides new support for ecological theories regarding species adaptability, distribution patterns, and environmental influences, and offers directions for future research.

## 1. Introduction

In ecological research, the spatial patterns of species distribution have long been a key topic for understanding biodiversity and ecosystem function [1]. The global distribution of species is influenced not only by their biological characteristics but also by environmental factors [1,2]. Parasitism, as a critical component of ecosystem functioning, is one of the most common life strategies among the species existing on Earth [3]. Parasites account for at least half of species diversity [4], with common examples including endoparasites (such as tapeworms, bacteria, and viruses), ectoparasites (such as fleas, lice, and other insects), parasitic fungi, and parasitic plants [5]. Among all parasitic organisms, the thousands of known parasitic plants occupy a particularly unique position [6]. Not only do they have distinct ecological roles in influencing the growth and physiology of their host plants, but they also exemplify extreme evolutionary adaptations of parasitic organisms [6]. For holoparasitic plants, such as the genus *Cuscuta* (Solanales Juss. ex Bercht. & J. Presl; Convolvulaceae Juss.; *Cuscuta* L.) [7], their parasitic traits, ecological adaptability, and growth characteristics provide an ideal system for exploring the driving mechanisms of biological distribution. While studies have revealed the relationship between the host niche breadth and the distribution range of parasitic organisms (e.g., fleas and worms) [8], such relationships remain largely enigmatic for parasitic plants. As a result, research on the relationships between the growth characteristics, ecological niches, and distribution patterns of parasitic plants is still relatively limited, particularly among closely related species, where comparative studies are notably scarce.

*Cuscuta* comprises four subgenera, among which species of the subg. *Grammica* are almost entirely reliant on host plant resources for growth and reproduction [9,10]. These species exhibit a high degree of specificity in terms of ecological niche and environmental selection [11,12]. In this study, we focus on three species within China that have been clearly recorded: *Cuscuta chinensis* Lam., *Cuscuta campestris* Yunck., and *Cuscuta australis* R.Br. (http://www.efloras.org), which are closely related species within subg. *Grammica*. Generally, these species grow on their hosts without roots or leaves, with only stem tissue present prior to flowering and fruiting [9]. As they are incapable of performing effective photosynthesis, they rely entirely on the host’s nutrients, leading to a significant reduction in global crop yields [12,13,14], and are common and harmful in many regions globally, with a broad host range including plants from the Fabaceae, Asteraceae, and other plant families [9]. Notably, these species not only reproduce on crops but also on weeds [9,10,15]. Therefore, even in the absence of host crops, weeds can contribute to the soil seed bank of these parasitic plants, thereby infecting subsequent crops. To date, no effective methods have been developed to control subg. *Grammica* species in agricultural settings, necessitating integrated management strategies [15,16]. Interestingly, these three species of subg. *Grammica* share certain ecological and biogeographical similarities, providing a valuable research opportunity to explore the relationship between growth characteristics, ecological niches, and the global distribution patterns of parasitic species. Investigating the growth rates, niche breadths, and global distribution of these species will not only help to understand their adaptive evolutionary patterns but also shed light on how parasitic plants respond to global changes and environmental shifts.

In this study, we focus on four key variables: growth rate, niche breadth, the number of global occurrence points, and global distribution area. Growth rate is a critical indicator of a species’ potential for growth in a specific environment. For parasitic plants, it reflects the efficiency with which they rely on host plants and the physiological constraints on their growth [17]. Niche breadth is an important ecological concept used to measure a species’ adaptability and the diversity of resources it utilizes, determining the range of environments to which the species can adapt [18]. Finally, the number of global occurrence points is a significant reflection of a species’ distribution, often closely related to its adaptability and ecological expansion capacity [19]. We hypothesize that these variables not only reflect the growth potential and ecological adaptability of each species but are also closely linked to their global distribution area.

The study organisms selected are three closely related holoparasitic plants: *C. chinensis*, *C. campestris*, and *C. australis*. These species exhibit certain biological and ecological similarities, while also displaying significant differences in their global distribution ranges [9]. The selection of these species provides a unique opportunity to examine whether growth rate, niche breadth, and the number of global occurrence points play significant roles in shaping their global distribution areas. We aim to reveal the potential impacts of these variables on species distribution and to explore their implications at both evolutionary and ecological levels.

Thus, the central question of this study is: For closely related holoparasitic plants in subg. *Grammica*, do growth rate, niche breadth, and the number of global occurrence points significantly influence their global distribution area? Based on this question, we mainly propose the following two hypotheses: (1) Growth rate is positively correlated with global distribution area; parasitic species with higher growth rates may be more capable of expanding across broader geographic regions; (2) niche breadth is positively correlated with global distribution area; parasitic species with greater ecological adaptability are likely to thrive and expand in a wider range of environments. By testing these hypotheses, we aim to provide new insights into the ecological mechanisms underlying the distribution of parasitic plants and offer valuable perspectives for species distribution studies in similar ecosystems.

## 2. Materials and Methods

### 2.1. Experimental Set Up

The experimental subjects of this study are three species of holoparasitic plants from subg. *Grammica* that have been widely distributed globally and are currently known in China: *C. chinensis*, *C. campestris*, and *C. australis*. Greenhouse experiments were designed for these three species of subg. *Grammica* and their respective hosts. Specifically, *C. chinensis*, *C. campestris*, and *C. australis* were selected as the parasitic species, while four pairs of closely related plant species were chosen as host plants. It is important to note that the criteria for selecting host plants were based on the condition that, for each host species pair, at least one host species had been observed in the wild to be parasitized by at least one of the three subg. *Grammica* species (*C. chinensis*, *C. campestris*, or *C. australis*). The four host species pairs include both native species from China and non-native American species (see Table 1 for details). The classification of subg. *Grammica* species was based on the Flora of China Database (http://www.efloras.org), selecting all three species of subg. *Grammica* clearly recorded in China for the parasitism experiments. The classification of the four closely related host species pairs (native and non-native American species, as shown in Table 1) was based on the Flora of China Database (http://www.efloras.org) and the “Checklist of Chinese Naturalized Plants” [20,21].

The experimental design is as follows: 3 species of subg. *Grammica* × 8 host species × 6 replicates + 2 control groups × 8 host species × 6 replicates = 240 pots. The experiment includes the following treatments: uninfected host plants (2 control groups), host plants infected by *C. chinensis*, host plants infected by *C. campestris*, and host plants infected by *C. australis*. A randomized complete block design (RCBD) was employed to arrange the experiment, with each treatment replicated 6 times. Data collected from these replicates were combined for analysis. For further details on seed collection and experimental procedures, please refer to Supplementary Section S1.

### 2.2. Growth Rate Data and Experimental Analysis

At the start of the experiments for the eight host species (t1, when species of subg. *Grammica* formed connections with the host xylem and phloem, and the haustoria showed noticeable swelling), the whole plant biomass of six uninfected control host plants was harvested to obtain the original host biomass (w1) and dried at 85 °C for at least 48 h to determine the dry weight. On day 60 post-inoculation (t2), the whole plant biomass of *E. heterophyllum* and *A. adenophora*, *S. decurrens* and *S. canadensis*, and *P. acinosa* and *P. americana* (one uninfected control group and three infected groups) was harvested, along with the corresponding biomass of the subg. *Grammica* species. Due to longer adaptation buffering following parasitism, subg. *Grammica* species on *B. pilosa* hosts experienced a prolonged adjustment period. Therefore, at 90 days post-infection (t3), the whole plant biomass of *B. biternata* and *B. pilosa* (one uninfected control group and three infected groups) was harvested, along with the biomass of the subg. *Grammica* species. The harvested host and subg. *Grammica* plant tissues were dried at 85 °C for at least 48 h to determine the dry weight. The daily average growth rate of the three subg. *Grammica* species (*C. chinensis*, *C. campestris*, and *C. australis*) was calculated as the mean growth rate of each species parasitizing the eight host species. Specifically, for each subg. *Grammica* species, the biomass produced by subg. *Grammica* species parasitizing the eight host species was first quantified. Secondly, based on the different experimental durations for each host, the daily average growth rate of subg. *Grammica* species parasitizing each host was calculated. Finally, the daily average growth rate of *C. chinensis*, *C. campestris*, and *C. australis* was determined by calculating the mean of the daily average growth rates for each species parasitizing the eight host species.

In addition to the statistical analysis of the growth rates of the three subg. *Grammica* species, this study also provides an overview of the experiment to thoroughly demonstrate its reliability. Specifically, the study reports the trends in the total biomass of *C. chinensis*, *C. campestris*, and *C. australis* when parasitizing eight different host species. It also presents the overall biomass produced by these species when parasitizing non-native American hosts and native hosts, respectively. Additionally, the relative total biomass of uninfected (control) and infected host groups is presented, illustrating the experimental outcomes. The relative total biomass of the host was calculated by subtracting the original host biomass (w1) from the total biomass at the end of each host trial.

### 2.3. Global Occurrence Data

The distribution information of *C. chinensis*, *C. campestris*, and *C. australis* species worldwide was obtained through two main sources: the GBIF database (https://www.gbif.org/) and field surveys. It is worth noting that species within subg. *Grammica* are frequently misidentified in Asia [22]. To mitigate potential classification errors, we conducted extensive field surveys in China from July 2021 to August 2023 to address any misidentification issues. The survey covered 22 provinces, 4 autonomous regions, and 3 municipalities directly under the central government (for details, see Supplementary Section S2). The occurrence data for *C. chinensis*, *C. campestris*, and *C. australis* collected through these field surveys will replace data from other sources for the Chinese region (this dataset will be published alongside other survey results; Quanzhong Zhang et al., in preparation). On a global scale, all collected occurrence data for subg. *Grammica* species were processed by removing duplicate records, low-accuracy records, outliers (e.g., records with both latitude and longitude of 0°), and erroneous records (such as those located on the sea or lake surface due to positional errors).

### 2.4. Niche Breadth Data

The 19 bioclimatic variables (BIO1-BIO19) and altitude environmental factor data (Table S1) were downloaded from the WorldClim database (https://www.worldclim.org/), with a spatial resolution of 2.5 arcminutes. This study used the “vegan” package in R version 4.2.1 [23] to perform PCA [24] and calculate the ecological niche breadth of *C. chinensis*, *C. campestris*, and *C. australis* species in the environmental factor space. Similar to the method employed by Kambach et al. (2019), the environmental factors included bioclimatic variables (BIO1–BIO19) and altitude, aimed at describing the diversity of species’ environmental adaptability [25,26]. During the analysis, the raw data were standardized to eliminate the influence of different environmental factor units on the results. The ecological niche breadth for each species was estimated by calculating the range of their point distributions within the PCA space (the extreme values of each principal component axis). For ease of comparison, we adopted a more straightforward approach, using the sum of the range differences for each principal component axis to represent the niche breadth index of each species. The results were visualized using bar charts. It is important to note that, for parasitic species, the host is also a critical factor influencing their survival [8]. In this study, an attempt was made to gather global host species data for the three subg. *Grammica* species. However, during the data processing process, it was found that there are limited host data available for *C. australis* in its major distribution areas such as Europe and Australia, which prevents a comprehensive representation of the host background of *C. australis* globally. When conducting comparative studies on *C. chinensis*, *C. campestris*, and *C. australis*, these missing data cannot be overlooked, as it may lead to inevitable biases and erroneous conclusions. Therefore, host ecological niches were not included in the calculation of the ecological niche breadth in this study.

### 2.5. Global Distribution Area Data of Species

To more effectively calculate and compare the global distribution areas of *C. chinensis*, *C. campestris*, and *C. australis*, a rigorous buffer zone statistical method was employed [27]. For the occurrence datasets of *C. chinensis*, *C. campestris*, and *C. australis*, a 1000 m buffer zone was constructed around each occurrence point using ArcGIS 10.2 [28] software to estimate their global distribution areas. During the data processing, to minimize the impact of local sampling effort and biases on area statistics, adjacent buffer zones were merged to prevent the overlapping areas from being counted multiple times. The geographic coordinate system WGS 1984 and the equal-area projection system Albers Equal Area Conic were used as reference systems, and the global distribution areas of the species were subsequently calculated.

### 2.6. Statistical Analysis Methods

In the experimental analysis, one-way analysis of variance (ANOVA) [29] was used to assess the significance of the relationship between the total biomass produced by *C. chinensis*, *C. campestris*, and *C. australis* when parasitizing eight different host species. Specifically, when the total biomass data for the three groups of subg. *Grammica* species met the conditions of normality and homogeneity of variance, Tukey’s HSD post-hoc multiple comparison test was used for further evaluation. Conversely, if at least one group of data did not meet the conditions of normality or homogeneity of variance, then the Kruskal–Wallis [30] non-parametric ANOVA was applied for significance testing, and the Bonferroni correction method was used to adjust the significance values. Independent samples *t*-tests [31] were used to assess the relationship between the total biomass produced by *C. chinensis*, *C. campestris*, and *C. australis* when parasitizing non-native and native host species, as well as the relationship between the relative total biomass of host species in the uninfected (control) and infected groups. Normality and homogeneity of variance tests were performed for each dataset. If the data were normally distributed and the variances were homogeneous, then independent samples *t*-tests were used to compare the mean differences between the two groups. In cases where the data were not normally distributed or the variances were not homogeneous, Welch’s *t*-test was applied to adjust for these issues and compare the mean differences. The significance level for all statistical tests was set at *p* < 0.05. The results are presented using bar charts.

To analyze the effects of growth rate, niche breadth, the number of global occurrence points, and global distribution area, the “ggpairs()” function in R version 4.2.1 [23] was used to construct a scatterplot matrix. This was followed by a comparative analysis of the performance of *C. chinensis*, *C. campestris*, and *C. australis* across these indicators, including growth rate, niche breadth, number of global occurrence points, and global distribution area.

## 3. Results

### 3.1. Parasitism Experiment Results

The results showed that the average growth rate of *C. chinensis* was 0.6034 mg/day, the average growth rate of *C. campestris* was 4.4124 mg/day, and the average growth rate of *C. australis* was 2.8282 mg/day. This suggests that the average growth rate of *C. campestris* on the eight host species (Table 1) is higher than that of *C. australis*, and in turn, the average growth rate of *C. australis* is higher than that of *C. chinensis*.

**Figure 1:**
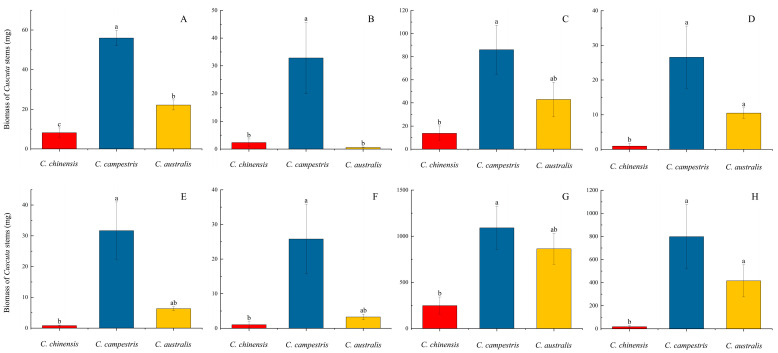
Stem biomass produced by *C. chinensis*, *C. campestris*, and *C. australis* parasitising eight different host species. Panels (**A**–**H**) represent the parasitism of the three *Cuscuta* species from the subg. *Grammica* on the corresponding host species, as follows: (**A**) *Bidens pilosa*; (**B**) *Bidens biternata*; (**C**) *Ageratina adenophora*; (**D**) *Eupatorium heterophyllum*; (**E**) *Solidago canadensis*; (**F**) *Solidago decurrens*; (**G**) *Phytolacca americana*; (**H**) *Phytolacca acinosa*. The significance level was set at *p* < 0.05. The lines in the bar charts represent the standard error of the mean (SEM) for each data group. The lowercase letters on each bar represent the significance between groups. If a bar shares the same letter with another bar, it indicates that there is no significant difference between them. If all the letters on one bar differ from those on another bar, it indicates a significant difference between the two.

Furthermore, we found that when *C. chinensis*, *C. campestris*, and *C. australis* parasitized eight different host species, respectively (Table 1), the total biomass of the three subg. *Grammica* species followed the pattern *C. campestris* > *C. australis* > *C. chinensis* (Figure 1). This may suggest differences in the parasitic abilities of the species within subg. *Grammica*. The results of the analysis of variance (Figure 1; Table S2) indicated that the biomass difference between *C. campestris* and *C. chinensis* was significant, and the differences between *C. campestris* and *C. australis*, as well as between *C. australis* and *C. chinensis*, were also relatively significant. Except for parasitism on *B. biternata*, *C. australis* generally showed higher biomass than *C. chinensis* (Figure 1).

**Figure 2:**
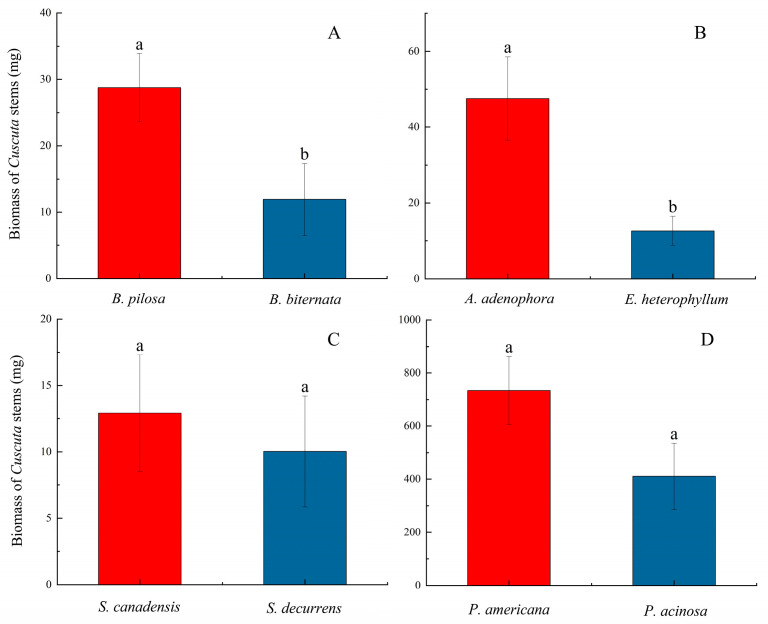
Total biomass of subg. *Grammica* species parasitizing non-native American hosts and native hosts from China. (**A**) represents the species pair *B. pilosa* and *B. biternata*; (**B**) represents the species pair *A. adenophora* and *E. heterophyllum*; (**C**) represents the species pair *S. canadensis* and *S. decurrens*; (**D**) represents the species pair *P. americana* and *P. acinosa*. The red bar represents the total biomass of subg. *Grammica* species parasitizing non-native American hosts. The blue bar represents the total biomass of subg. *Grammica* species parasitizing native hosts. Statistical significance was set at *p* < 0.05. The lines in the bar charts represent the standard error of the mean (SEM) for each group of data. The lowercase letters on each bar represent the significance between groups. If a bar shares the same letter with another bar, it indicates that there is no significant difference between them. If all the letters on one bar differ from those on another bar, it indicates a significant difference between the two.

**Figure 3:**
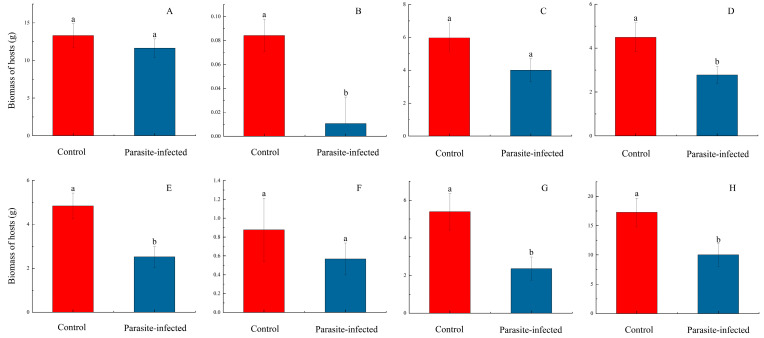
Relative total biomass of hosts in the uninfected (control) and infected groups. Panels (**A**–**H**) represent 8 host species, as follows: (**A**) *B. pilosa*; (**B**) *B. biternata*; (**C**) *A. adenophora*; (**D**) *E. heterophyllum*; (**E**) *S. canadensis*; (**F**) *S. decurrens*; (**G**) *P. americana*; (**H**) *P. acinosa*. The red bar represents the relative total biomass of hosts in the uninfected (control) group. The blue bar represents the relative total biomass of hosts in the infected group. Statistical significance was set at *p* < 0.05. The lines in the bar charts represent the standard error of the mean (SEM) for each group of data. The lowercase letters on each bar represent the significance between groups. If a bar shares the same letter with another bar, it indicates that there is no significant difference between them. If all the letters on one bar differ from those on another bar, it indicates a significant difference between the two.

For the eight host plant species (four species pairs, including both non-native and native hosts), the total biomass produced by the three subg. *Grammica* species following parasitism showed a pattern where the biomass produced when parasitizing non-native American host species was greater than that produced when parasitizing native host species (Figure 2; Table S3). Specifically, significant differences were observed between *B. pilosa* and *B. biternata*, as well as between *A. adenophora* and *E. heterophyllum*. Statistical comparisons of the relative total biomass between uninfected (control) and infected host groups revealed that, in general, the biomass of hosts was significantly reduced after parasitism (Figure 3; Table S4).

### 3.2. Niche Breadth and Global Distribution Area

The principal component loading statistics (Table S5) indicate that for *C. chinensis*, variables such as BIO1 (Annual mean temperature), BIO6 (Min temperature of the coldest month), BIO9 (Mean temperature of the driest quarter), and BIO11 (Mean temperature of the coldest quarter) have high positive loadings on the PC1 axis. In contrast, BIO5 (Max temperature of the warmest month) shows a large negative loading on the PC2 axis. These results suggest that temperature is a key factor influencing the growth of *C. chinensis*, which tends to favor milder climates and may be better suited to colder regions with more stable temperatures.

For *C. australis* (Table S6), the PC1 axis shows relatively large positive loadings for BIO4 (Temperature seasonality) and BIO7 (Temperature annual range), indicating that temperature seasonality and annual temperature variation significantly affect the species. *C. australis* is likely more adaptable to seasonal or annual fluctuations in temperature compared to *C. chinensis*. The relatively large negative loadings on BIO6 (Min temperature of the coldest month) and BIO9 (Mean temperature of the driest quarter) may also suggest that *C. australis* prefers colder winter environments. On the PC2 axis, the larger positive loadings of BIO8 (Mean temperature of the wettest quarter), BIO13 (Precipitation of the wettest month), and BIO16 (Precipitation of the wettest quarter) suggest that seasonal temperature and precipitation during the summer have a significant influence on the distribution of *C. australis*.

Finally, for *C. campestris* (Table S7), the PC1 axis is primarily influenced by factors such as BIO1 (Annual mean temperature), BIO6 (Min temperature of the coldest month), BIO11 (Mean temperature of the coldest quarter), and BIO4 (Temperature seasonality), indicating that *C. campestris* may be more adaptable to environments with larger temperature differences and colder temperatures. On the PC2 axis, there are significant positive loadings, especially for BIO12 (Annual precipitation), BIO14 (Precipitation of the driest month), and BIO17 (Precipitation of the driest quarter), suggesting that *C. campestris* is also well-suited to environments with relatively high precipitation in winter and throughout the year, as well as those with seasonal wet–dry transitions.

To more comprehensively reflect the position and adaptability of species in ecological space, this study calculates the niche breadth using all principal components. The total variance of the entire dataset and the collective contribution of all principal components explain the ecological niches of the species. This approach aids in understanding the species’ adaptability to subtle environmental changes (through principal components with low variance).

**Figure 4:**
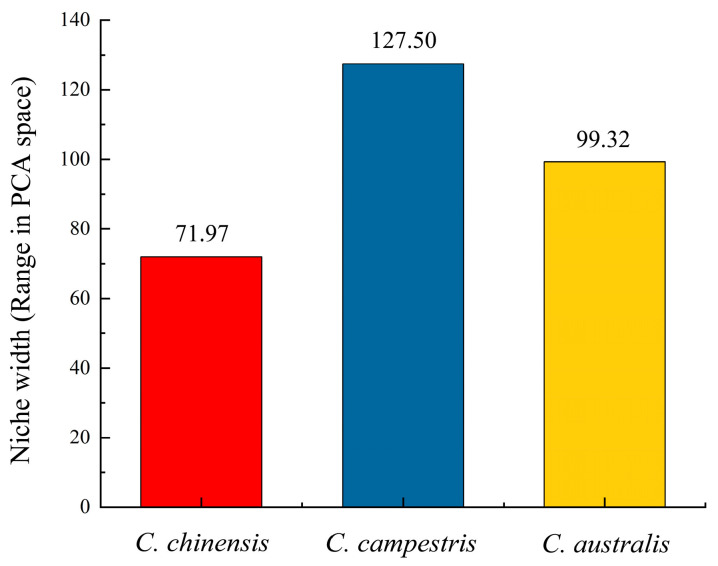
Niche breadth of *C. chinensis*, *C. campestris*, and *C. australis* in the environmental factor PCA space.

The statistics (Tables S8–S10) show that the first 10 principal components of *C. chinensis* explain 99.61% of the total variance. The first 10 principal components of *C. campestris* explain 99.23% of the total variance. The first 10 principal components of *C. australis* explain 99.37% of the total variance. According to the calculations (Table S11), as shown in Figure 4, the niche breadth index of *C. campestris* is higher than that of *C. australis*, while the niche breadth index of *C. australis* is higher than that of *C. chinensis*. The differences in the niche breadth indices among the three subg. *Grammica* species may suggest variations in the environmental adaptability of the subg. *Grammica* species.

The point data for the species of the subg. *Grammica* were processed, revealing that there are 363 global occurrence points for *C. chinensis*, 7450 global occurrence points for *C. campestris*, and 607 global occurrence points for *C. australis*. After buffering, the calculated results show that the global distribution area of *C. chinensis* is approximately 555.69 km^2^, *C. campestris* covers approximately 18,596.3 km^2^, and *C. australis* occupies approximately 1674.61 km^2^ globally.

### 3.3. The Relationships Between the Variables

**Figure 5:**
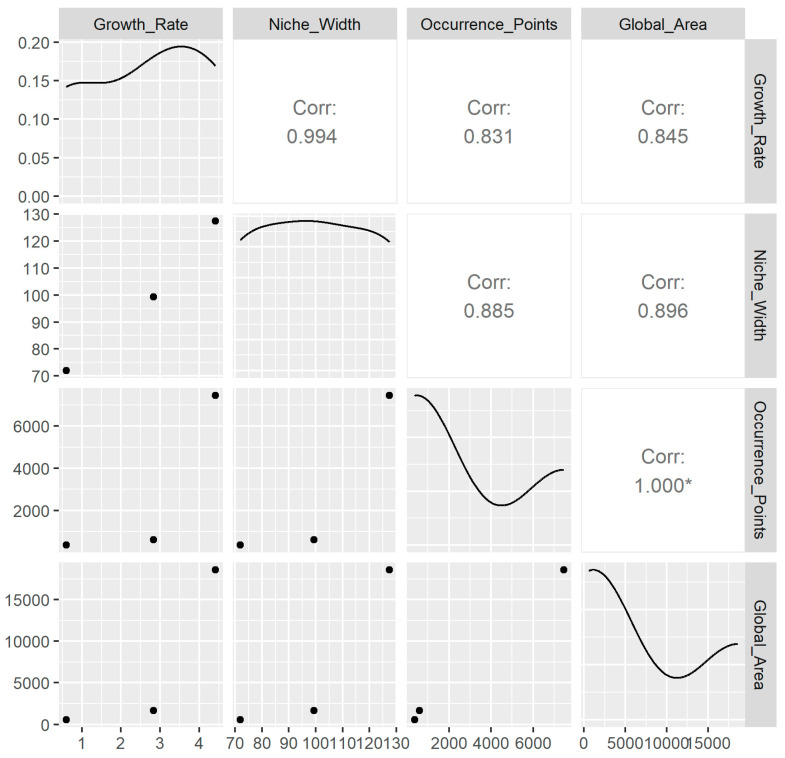
Scatterplot matrix showing the relationships between growth rate, niche breadth, number of global occurrence points, and global distribution area for *C. chinensis*, *C. australis*, and *C. campestris*. And the asterisk represents a strong correlation.

We observed some interesting results from the scatterplot matrix of growth rate, niche breadth, number of global occurrence points, and global distribution area for *C. chinensis*, *C. campestris*, and *C. australis* (Figure 5). For the three subg. *Grammica* species, the correlation coefficient between growth rate and global distribution area is 0.845, indicating a significant positive correlation between these two variables. In other words, for these three subg. *Grammica* species, when the growth rate of a species increases, its global distribution area tends to increase as well, and vice versa. The correlation coefficient between niche breadth and global distribution area is 0.896, suggesting a significant positive correlation between these two variables as well. As niche breadth increases, global distribution area also tends to expand. The correlation coefficient between the number of global occurrence points and global distribution area is close to 1. This indicates that in this study, the number of global occurrence points of species in the subg. *Grammica* has a very strong influence on the global distribution area. This may suggest that *C. chinensis*, *C. campestris*, and *C. australis* are not locally or narrowly distributed worldwide, but instead are widely distributed across various environmental spaces.

In addition (Figure 5), the correlation coefficient between the growth rate and niche breadth for the three subg. *Grammica* species is 0.994, suggesting that the relationship between these two variables may be very strong. The correlation coefficient between growth rate and the number of global occurrence points is 0.831, indicating a strong positive correlation; as the growth rate increases, the number of global occurrence points also tends to increase. The correlation coefficient between niche breadth and the number of global occurrence points is 0.885, indicating a significant positive correlation as well; as niche breadth increases, the number of global occurrence points is also likely to increase.

## 4. Discussion

Parasites account for nearly half of the species diversity on Earth [4], yet parasitology still appears to be plagued by data limitations that are almost insurmountable [32,33], leaving us with little understanding of their biogeography and global distribution patterns. Subg. *Grammica* species, fully parasitic plants, serve as representatives of the extreme evolutionary adaptations found among thousands of parasitic plants. Their intrinsic growth patterns, niche characteristics, and distribution patterns provide an ideal system for studying the driving mechanisms behind the distribution of parasitic organisms. The key findings from this study’s experiments and analyses suggest that for subg. *Grammica* species, there is a strong positive correlation between growth rate, niche breadth, number of global occurrence points, and global distribution area (Figure 5). As growth rate increases, so do niche breadth, number of global occurrence points, and global distribution area. Notably, the correlation coefficient between growth rate and niche breadth is 0.994 (Figure 5), suggesting that although parasitic plants interact with the environment through hosts as intermediaries, there may still be a strong relationship between the intrinsic growth characteristics of parasitic plants and their external ecological niche.

In addition, *C. campestris* exhibits significantly higher growth rates on both non-native American and native hosts compared to *C. australis* and *C. chinensis*, and similar results can also be observed between *C. australis* and *C. chinensis* (Figure 1). We believe that the growth performance of these three species of subg. *Grammica* is likely regulated by their own genes and genetic traits. The entire experiment was conducted in a greenhouse with similar environmental conditions (see Supplementary Section S1), thus allowing us to rule out the influence of environmental factors such as temperature, humidity, and light conditions. Furthermore, when they parasitize non-native American hosts, they produce a higher biomass compared to when parasitizing native hosts (Figure 1 and Figure 2). Regarding this phenomenon, Hautier suggested that the growth rate and biomass of parasites increase with the host’s growth rate [34]. Parasitic plants grow more vigorously on non-native hosts [35], which can be explained by the fact that the host provides more resources [36]. This view may explain some of the results observed in this study. However, when parasitizing *P. americana* and *P. acinosa*, we observed an inconsistent pattern (Figure 3G,H). Within the same time frame, *P. acinosa* produced significantly more biomass than *P. americana* (Figure 3G,H), yet the biomass of the parasitic plants was the opposite (Figure 1G,H and Figure 2D). Therefore, this study presents another intriguing hypothesis: when *C. campestris*, *C. australis*, and *C. chinensis* parasitize non-native American hosts, they produce higher biomass compared to when parasitizing native hosts, possibly due to the long-term co-evolution between the parasitic species and their hosts [37], which may result in better adaptation to the physiological characteristics and ecological behaviors of these hosts. There is evidence to suggest that the center of biodiversity for species of subg. *Grammica* lies near Mexico in the Americas [9,10], and the distribution of closely related species to *C. campestris*, *C. australis*, and *C. chinensis* is also largely restricted to the Americas [10,22]. Therefore, it is possible that these three species of subg. *Grammica* have experienced a longer evolutionary history with American hosts. Of course, we still require more evidence to support this possibility.

As holoparasitic plants, subg. *Grammica* species can reproduce asexually through their young shoots [12,15]. Seed germination typically requires seasonal variations in temperature and precipitation, with seeds generally germinating during the transition between spring and summer and growing alongside host species until autumn [11,16]. If the host is a perennial species that continues to grow through the winter, subg. *Grammica* species are likely to persist on the host [11,16]. Therefore, this study selected 19 bioclimatic variables and altitude factors to estimate niche breadth, as temperature, precipitation, and their fluctuations are likely to be crucial (Tables S5–S7) for *subg. Grammica* species [13]. During the PCA analysis, 20 principal component axes were generated, ordered by the variance they explained. Notably, the distribution range of species along different principal component axes is regulated by the contribution and significance of each axis to the species’ ecological niche characteristics, with the range gradually decreasing as the explained variance of the axes diminishes (Table S11). The three subg. *Grammica* species together explained all the variance across the 20 principal component axes. Therefore, unlike previous studies that selected the first two principal component axes to calculate niche breadth [25], this study used the range of subg. *Grammica* species across all principal component dimensions to specifically quantify and compare the niche breadth index for the three species.

In a recent study, Cebrián-Camisón revealed a positive correlation between the host species abundance of parasites and the number of occurrence sites [38]. Species with broader ecological niches are typically able to survive under a wider range of environmental conditions and may have more occurrence sites [39], which aligns with our findings for *C. campestris*. Not only does *C. campestris* have the broadest ecological niche (127.50) among the three species of subg. *Grammica*, but it also has as many as 7450 occurrence points, suggesting that this species may have a high degree of adaptability and niche generalization on a global scale. Furthermore, the strong correlation between ecological niche breadth and global distribution area (Figure 5) may, from the perspective of parasitology, further support the “niche breadth-distribution area” hypothesis in modern ecology [40]. By utilizing a broader range of resources and maintaining viable populations under a wider set of conditions, species are expected to become more widespread, which will result in a positive correlation between ecological niche breadth and geographic range size [41]. Furthermore, species with broader ecological niches are more likely to possess a wide range of resources within that niche [40], such as host resources. It is noteworthy that during our attempt to gather host species data for the three species of subg. *Grammica* worldwide, we found that *C. campestris* has a host species richness far exceeding that of *C. australis* and *C. chinensis*. Although this aspect is not detailed in the main text, based on the available evidence, we can still hypothesize the following: For holoparasitic plants *C. chinensis*, *C. campestris*, and *C. australis*, there appears to be a positive correlation between growth rate, host richness, and ecological niche breadth. We urge researchers in the relevant fields to devote more attention to the classification and identification of hosts for *Cuscuta* L. species in future studies, to support more comprehensive and robust research.

Although the findings of this study provide evidence supporting the correlation between the growth rate, ecological niche breadth, number of occurrence points, and global distribution area of parasitic plants in subg. *Grammica*, there are still several points that warrant further exploration. Firstly, our study is based on data from three species within the subg. *Grammica* and does not delve into the growth performance differences of additional species within the subg. *Grammica* across different ecosystems. For instance, *C. australis* has a broad global distribution, but its growth rate may be significantly influenced by environmental conditions in various regions. Future research could involve selecting a broader range of species from the subg. *Grammica* to participate in experimental analyses across different ecological regions, further investigating the relationship between the growth rate of parasitic plants and their ecological niche. Secondly, although we attempted to correct the distribution locations of the three species in the subg. *Grammica* through field sampling and processed the global distribution data of subg. *Grammica* species by removing duplicate records, outliers, and incorrect coordinates, the remaining distribution data may still be influenced by sampling efforts and researcher biases. To address this issue, future research should incorporate as much independent and reliable distribution information as possible to more accurately represent the true abundance of species and assist researchers in better estimating the species’ distribution ranges. Lastly, the measurement of ecological niche breadth in the dataset is based on the species’ distribution characteristics in geographical space, without fully considering other dimensions of the niche, such as the diversity of host resource utilization [8]. Future studies will require more data on both abiotic and biotic factors to more precisely analyze the ecological relationships between species.

Specifically, the results of this study (Figure 5) indicate that *C. chinensis* exhibits a relatively low growth rate and a narrow ecological niche, with a more limited distribution range, suggesting that it may be a species more sensitive to environmental changes and with limited adaptability. *C. australis* has a moderate growth rate, a larger niche breadth, and a wider distribution area than *C. chinensis*, suggesting it is a species with moderate adaptability that can survive in a variety of environments. *C. campestris* has a high growth rate, the largest niche breadth, and the widest distribution area, demonstrating that it is a species highly adaptable to environmental changes and capable of thriving in multiple ecosystems.

Exploring the relationship between the growth rate of species and their adaptability, ecological niche, and distribution range is one of the key topics in ecological research [1,2]. This study provides new insights into this subject through an analysis of species within the holoparasitic plant subg. *Grammica*. Specifically, the strong correlation between the growth rate of parasitic plants and their global distribution area may reveal that the spatial distribution of parasitic organisms is influenced not only by local environmental factors but also by broader biogeographical forces. Moreover, the extensive ecological niche breadth of parasitic organisms offers a fresh perspective for understanding species’ performance across different ecosystems. In particular, when parasitic organisms exhibit a high growth rate, they may perform better in diverse environmental conditions, which is of significant theoretical importance for predicting their future adaptability on a global scale. By combining multiple ecological indicators, such as growth rate, ecological niche breadth, number of occurrence points, and global distribution area, this study presents a comprehensive profile of parasitic plant distribution and ecological adaptation, offering new approaches and methodologies for future research in ecology and biogeography.

## 5. Conclusions

This study provides an in-depth investigation into the complex relationships between growth rate, ecological niche breadth, and global distribution patterns in species of the holoparasitic plant subg. *Grammica*. Through data analysis, the study confirms significant correlations among these indicators. The main findings of the experiments and analysis suggest that for the holoparasitic plants *C. campestris*, *C. australis*, and *C. chinensis*, there is a strong positive correlation between growth rate, ecological niche breadth, number of global occurrence points, and global distribution area. The significant correlation between growth rate and ecological niche breadth suggests that the intrinsic growth characteristics of parasitic plants may significantly influence their realized ecological niche. Furthermore, the experimental results indicate that when *C. campestris*, *C. australis*, and *C. chinensis* parasitize non-native American hosts, they produce greater biomass than when parasitizing native hosts from China, implying that the diffusion history of subg. *Grammica* species in China may need to be reconsidered. In conclusion, this study offers new support for ecological theory and provides directions for future research, particularly in exploring species adaptability, distribution patterns, and their environmental influences.

## Figures and Tables

**Table 1:** The four host species pairs include both native and non-native American species.

Family	Native Host	Non-Native American Host
Asteraceae	*Bidens biternata* (Lour.) Merr. et Sherff	*Bidens pilosa* L.
Asteraceae	*Eupatorium heterophyllum* DC.	*Ageratina adenophora* (Sprengel) R. M. King & H. Robinson
Asteraceae	*Solidago decurrens* Lour.	*Solidago canadensis* L.
Phytolaccaceae	*Phytolacca acinosa* Roxb.	*Phytolacca americana*

## Data Availability

The data presented in this study are available as Supplementary Materials.

## Supplementary Materials

The following supporting information can be downloaded at: https://www.mdpi.com/article/10.3390/plants14060831/s1, Section S1. Supplementary information on experimental details; Section S2: Supplementary information on field survey; Section S3: This appendix includes the meanings of the environmental variable factors; detailed information of the statistical tests conducted in the experiment; the principal component loadings table generated during the calculation of niche breadth, PCA variance explained, and the width of three subg. Grammica species in the PCA space along each principal component axis.
