# Supplementary material for: Do Faster-Growing Holoparasitic Plant Species Exhibit Broader Niches and Wider Global Distributions?
Source: Plants (Basel). 2025 Mar 7;14(6):831. doi: 10.3390/plants14060831 (PMC11946478; doi:10.3390/plants14060831)
Supplement: Supplementary file 1 [file plants-14-00831-s001.zip › plants-3447696-supplementary.pdf]

plants

Do faster-growing holoparasitic plant species exhibit broader niches and wider global distributions?

Section S1. Supplementary information on experimental details.

Seeds of *Cuscuta* subg. *Grammica* species were collected from the field. Specifically, in November 2021, seeds of *C. chinensis* were collected from fields in Derong County, Ganzi Tibetan Autonomous Prefecture, Sichuan Province. In August 2022, seeds of *C. campestris* were collected from infected host plants along the roadside in Binchuan County, Dali Bai Autonomous Prefecture, Yunnan Province. In September 2022, seeds of *C. australis* were collected from fields in Pingluo County, Ningxia Hui Autonomous Region. Seeds of host species were obtained through field collection and market purchase. All seeds were stored at room temperature (22-25°C) prior to experimental use.

Four host pairs (Table 1) were cultivated from seeds, placed on the surface of agar in Petri dishes, and kept in a plant growth chamber at 25°C with a 12-hour light/dark cycle, awaiting seed germination. Once germination occurred, the host plants were transferred to plastic pots (20 cm in diameter) filled with soil untreated with herbicides. When the host seedlings were small, parasitism by subg. *Grammica* species could result in their death. During the experiment, when each host pair met the following two conditions: (1) the individuals of both host species being of comparable size in the above-ground parts, and (2) the individuals having grown sufficiently such that parasitism by subg. *Grammica* species would not cause immediate death, seedlings of subg. *Grammica* species (approximately 3–4 cm in length, germinated for about 2 days) were transferred to pots containing host plants, positioned about 2 cm from the host stem. The germination process for *C. chinensis*, *C. campestris*, and *C. australis* seeds was as follows: The seeds were first placed on the surface of agar in Petri dishes, which were then placed in a plant growth chamber at 25°C with a 12-hour light/dark cycle, awaiting germination of *C. chinensis*, *C. campestris*, and *C. australis*. Subsequent experiments were conducted in a greenhouse, where the indoor temperature ranged approximately from 15°C to 30°C during the trial period.

At the beginning of the experiment, due to differences in the time required for the various hosts to reach the stage of parasitism from seedling growth in the experimental soil, the timing of parasitism initiation by subg. *Grammica* species varied for each host. However, for each pair of host species within the same species group, the experimental period following the onset of parasitism remained consistent. Specifically, the experiment periods following parasitism for the host pairs *E. heterophyllum* and *A. adenophora*, *S. decurrens* and *S. canadensis*, and *P. acinosa* and *P. americana* were all 60 days, whereas for *B. biternata* and *B. pilosa*, the experimental period after parasitism lasted 90 days. The entire parasitism experiment started on 11th May 2023 and ended on 23rd December 2023. The start and end dates for each of the eight host parasitism experiments were as follows: *B. pilosa* (11th May 2023–8th August 2023); *B. biternata* (11th May 2023–8th August 2023); *P. americana* (11th May 2023–9th July 2023); *P. acinosa* (4th June 2023–2nd July 2023); *A. adenophora* (8th September 2023–6th November 2023); *E. heterophyllum* (17th August 2023–15th October 2023); *S. canadensis* (14th July 2023–11th September 2023); *S. decurrens* (26th October 2023–24th December 2023).

For each host, at the beginning of the experiment, seedlings of three subg. *Grammica* species (*C. chinensis*, *C. campestris*, and *C. australis*) were placed approximately 2 cm away from the host stem. Only one seedling was placed at a time to ensure successful parasitism by a single subg. *Grammica* individual. The start date of each host experiment was the date

when a subg. *Grammica* seedling first successfully parasitized the first experimental pot of that host (evidenced by the clear swelling of the haustorium and the connection formed between the subg. *Grammica* species and the host's xylem and phloem). The end date of each experiment was when the final biomass of the subg. *Grammica* species and its host was harvested. During the experiment, daily watering was performed to maintain soil moisture, with specific amounts adjusted depending on the clarity of the weather.

Section S2. Supplementary information on field survey.

Based on specimen records, image records, or sampling locations provided by the Chinese Virtual Herbarium (CVH, https://www.cvh.ac.cn/), the National Specimen Information Infrastructure (NSII, http://nsii.org.cn/), the Plant Photo Bank of China (PPBC, https://ppbc.iplant.cn/), the Global Biodiversity Information Facility (GBIF, https://www.gbif.org/), and the relevant literature, we have compiled all recorded occurrence points of subg. *Grammica* species in China. During the field survey, this study visited nearly all recorded occurrence points of subg. *Grammica* species across 22 provinces, 4 autonomous regions, and 3 municipalities directly under the central government. The surveyed areas specifically included the provinces of Heilongjiang, Jilin, Liaoning, Hebei, Shanxi, Shandong, Henan, Jiangsu, Zhejiang, Anhui, Jiangxi, Fujian, Hubei, Hunan, Guangdong, Sichuan, Guizhou, Yunnan, Shaanxi, Gansu, Qinghai, and Taiwan; the autonomous regions of Guangxi, Inner Mongolia, Ningxia, and Xinjiang; and the municipalities of Beijing, Tianjin, and Chongqing. This dataset will be published alongside other survey results (Quanzhong Zhang et al., currently in preparation).

Section S3. This appendix includes the meanings of the environmental variable factors; detailed information of the statistical tests conducted in the experiment; the principal component loadings table generated during the calculation of niche breadth, PCA variance explained, and the width of three subg. *Grammica* species in the PCA space along each principal component axis.

Table S1. The meaning of environmental variable factors.

Abbreviation of environmental variables	Name of environmental variables
BIO1	Annual Mean Temperature
BIO2	Mean Diurnal Range (Mean of monthly (max temp - min temp))
BIO3	Isothermality (BIO2/BIO7) (×100)
BIO4	Temperature Seasonality (standard deviation ×100)
BIO5	Max Temperature of Warmest Month
BIO6	Min Temperature of Coldest Month
BIO7	Temperature Annual Range (BIO5-BIO6)
BIO8	Mean Temperature of Wettest Quarter
BIO9	Mean Temperature of Driest Quarter
BIO10	Mean Temperature of Warmest Quarter
BIO11	Mean Temperature of Coldest Quarter
BIO12	Annual Precipitation
BIO13	Precipitation of Wettest Month
BIO14	Precipitation of Driest Month
BIO15	Precipitation Seasonality (Coefficient of Variation)

BIO16	Precipitation of Wettest Quarter
BIO17	Precipitation of Driest Quarter
BIO18	Precipitation of Warmest Quarter
BIO19	Precipitation of Coldest Quarter
EL	Altitude

Table S2. Analysis of variance (ANOVA) statistical test table.

	H Value	F Value	DF (Between Groups)	DF (Within Groups)	p-value	Post-Hoc Test
B. pilosa		66.739	2	15	0.000	Tukey HSD: Group 2 > Group 3 (p = 0.000); Group 2 > Group 1 (p = 0.000); Group 3 > Group 1 (p = 0.013)
B. biternata	10.918		2		0.004	Bonferroni: Group 2 > Group 1 (p = 0.022); Group 2 > Group 3 (p = 0.008); Group 1 > Group 3 (p = 1.000)
A. adenophora	8.222		2		0.016	Bonferroni: Group 2 > Group 3 (p = 0.529); Group 2 > Group 1 (p = 0.012); Group 3 > Group 1 (p = 0.390)
E. heterophyllum	12.066		2		0.002	Bonferroni: Group 2 > Group 3 (p = 1.000); Group 2 > Group 1 (p = 0.003); Group 3 > Group 1 (p = 0.032)
S. canadensis	15.221		2		0.000	Bonferroni: Group 2 > Group 3 (p = 0.153); Group 2 > Group 1 (p = 0.000); Group 3 > Group 1 (p = 0.153)
S. decurrens	9.697		2		0.008	Bonferroni: Group 2 > Group 3 (p = 0.455); Group 2 > Group 1 (p = 0.006); Group 3 > Group 1 (p = 0.281)
P. americana	8.784		2		0.012	Bonferroni: Group 2 > Group 3 (p = 1.000); Group 2 > Group 1 (p = 0.015); Group 3 > Group 1 (p = 0.080)
P. acinosa	11.661		2		0.003	Bonferroni: Group 2 > Group 3 (p = 1.000); Group 2 > Group 1 (p = 0.004); Group 3 > Group 1 (p = 0.024)

Note: The post-hoc test p-values from the Kruskal–Wallis one-way ANOVA analysis are the values adjusted using the Bonferroni correction method; Group 1 represents six replicates of *C. chinensis*, Group 2 represents six replicates of *C. campestris*, and Group 3 represents six replicates of *C. australis*. The significance level was set at $P < 0.05$.

Table S3. Statistical test table for the significance of the relationship between the total biomass produced by subg. *Grammica* species parasitizing non-native American and native species.

	Welch's t-value	DF	p-value	Mean (mg) (Group 1)	Standard Error (mg) (Group 1)	Mean (mg) (Group 2)	Standard Error (mg) (Group 2)
B. pilosa and *B. biternata*	2.26	33.90	0.03	28.77	5.13	11.91	5.42
A. adenophora and *E. heterophyllum*	2.98	21.07	0.007	47.54	11.04	12.67	3.85
S. canadensis and *S. decurrens*	0.48	33.92	0.636	12.93	4.39	10.03	4.18
P. americana and *P. acinosa*	1.81	33.97	0.08	734.03	128.43	410.71	124.64

Note: Group 1 refers to subg. *Grammica* species parasitizing non-native American hosts; Group 2 refers to subg. *Grammica* species parasitizing native hosts. The significance level was set at $P < 0.05$.

Table S4. Statistical test table for the significance of the relationship between the relative total biomass of host species in the uninfected (control) and infected groups.

	t-value	Welch's t-value	DF	p-value	Mean (g) (Control Group)	Standard Error (g) (Control Group)	Mean (g) (Parasite-infected Group)	Standard Error (g) (Parasite-infected Group)
B. pilosa	0.72		22	0.478	13.31	1.57	11.64	1.22
B. biternata		2.82	21.73	0.009	0.08	0.01	0.01	0.02
A. adenophora	1.47		22	0.155	5.97	0.91	4.00	0.70
E. heterophyllum	2.20		22	0.039	4.50	0.66	2.78	0.39
S. canadensis	2.56		22	0.018	4.84	0.59	2.52	0.48
S. decurrens	0.88		22	0.386	0.88	0.34	0.57	0.17
P. americana		2.63	9.35	0.027	5.39	0.97	2.37	0.61
P. acinosa		2.35	11.93	0.037	17.28	2.42	10.01	1.92

Note: The significance level was set at $P < 0.05$.

Table S5. Principal component loadings table for *C. chinensis*.

	PC1	PC2	PC3	PC4	PC5	PC6	PC7	PC8	PC9	PC10
BIO1	0.3080	-0.1846	-0.0360	0.0947	-0.0155	0.0078	-0.0364	-0.0781	-0.1080	-0.0232
BIO2	-0.1514	-0.2936	0.0756	0.0586	0.5058	-0.5907	-0.0873	-0.2311	0.0376	0.1789
BIO3	0.2582	-0.0762	0.2364	0.2529	0.1574	-0.2864	0.0549	0.4081	0.4926	0.0992
BIO4	-0.2936	-0.0347	-0.2549	-0.1882	0.0392	-0.0040	-0.0113	0.1083	-0.0263	-0.0494
BIO5	0.1408	-0.3382	-0.2976	-0.0551	0.1815	-0.0185	-0.0545	-0.1204	-0.2383	-0.0036
BIO6	0.3283	-0.0541	0.0672	0.1417	-0.0734	0.0924	-0.0203	-0.0611	-0.0525	0.0215
BIO7	-0.2900	-0.1058	-0.2185	-0.1817	0.1687	-0.1100	-0.0045	0.0081	-0.0588	-0.0253
BIO8	0.1654	-0.3061	-0.2769	-0.0539	-0.1682	-0.1238	-0.1861	0.2299	0.3026	-0.6347
BIO9	0.3142	-0.1134	0.0779	0.1516	0.0644	0.0746	0.0000	-0.1701	-0.2865	0.1377
BIO10	0.1919	-0.2987	-0.2996	-0.0487	0.0237	0.0503	-0.0393	-0.0567	-0.1820	-0.0585
BIO11	0.3231	-0.0945	0.0876	0.1382	-0.0257	0.0224	-0.0180	-0.1080	-0.0573	0.0105
BIO12	0.2444	0.2394	-0.0127	-0.3024	0.1768	-0.0589	-0.1217	-0.0102	0.0180	-0.0292
BIO13	0.2083	0.0798	0.1343	-0.4932	0.2723	0.1467	-0.1324	0.0733	0.1098	0.0115
BIO14	0.1249	0.3250	-0.3179	0.0992	0.1109	-0.0242	0.1763	-0.4526	0.3400	-0.1006
BIO15	0.0503	-0.2333	0.2895	-0.3555	-0.1645	-0.1795	0.7721	-0.1557	-0.0334	-0.2139
BIO16	0.2302	0.0988	0.1223	-0.4625	0.2163	0.0820	-0.1378	0.0270	0.0192	0.0299
BIO17	0.1357	0.3328	-0.3045	0.1010	0.1367	-0.0881	0.1863	-0.2535	0.1432	-0.1044
BIO18	0.1365	0.2726	-0.0119	-0.1808	-0.5120	-0.6529	-0.2715	-0.0652	-0.2293	0.0606
BIO19	0.1498	0.2981	-0.2448	0.1282	0.2738	-0.1579	0.3500	0.5647	-0.4360	-0.0638
EL	-0.1286	0.1793	0.4247	0.2016	0.2641	-0.0248	-0.1961	-0.1567	-0.2790	-0.6726

Table S6. Principal component loadings table for *C. australis*.

	PC1	PC2	PC3	PC4	PC5	PC6	PC7	PC8	PC9	PC10
BIO1	-0.2849	0.2377	0.1649	0.0468	0.0097	-0.0617	-0.1038	0.0207	-0.0209	0.0667
BIO2	-0.0012	-0.0122	0.3481	-0.3602	-0.4802	-0.1529	0.5498	-0.1533	0.0497	0.0762
BIO3	-0.3097	0.0982	0.0687	-0.3047	0.0426	-0.1550	0.2833	0.1569	-0.2180	-0.1775
BIO4	0.3505	-0.0267	-0.0001	0.1086	-0.1896	0.1203	-0.1038	-0.0022	-0.0534	-0.0111
BIO5	-0.0701	0.2455	0.3443	0.1375	-0.3632	0.2564	-0.1637	-0.2216	0.0189	-0.1122
BIO6	-0.3467	0.1095	0.0436	0.0274	0.1469	-0.0104	-0.0759	-0.0596	0.0442	0.0234
BIO7	0.3409	-0.0238	0.0831	0.0226	-0.2920	0.1073	0.0189	-0.0200	-0.0398	-0.0669
BIO8	0.0558	0.3744	0.1267	0.1381	-0.0777	-0.4671	-0.0578	0.5057	0.2125	-0.0938
BIO9	-0.3391	-0.0331	0.0863	-0.0005	0.0801	0.2518	-0.0161	-0.3585	-0.1327	0.1919
BIO10	-0.0505	0.3399	0.2623	0.2314	-0.2182	0.0939	-0.3101	-0.0136	-0.0474	0.0611
BIO11	-0.3361	0.1439	0.0934	-0.0154	0.0952	-0.0702	-0.0241	-0.0094	0.0264	0.0420
BIO12	-0.0074	0.3422	-0.3240	-0.1117	-0.0317	0.0716	0.0357	-0.1143	0.1792	-0.1725
BIO13	0.1481	0.3760	-0.1371	-0.1174	0.1420	0.1640	0.1485	-0.1701	-0.0014	-0.3626
BIO14	-0.1483	0.0102	-0.4109	0.0487	-0.3724	-0.2078	-0.0829	-0.0672	-0.4335	0.0182
BIO15	0.2321	0.2584	0.1623	-0.1585	0.2098	0.1582	0.0154	0.2690	-0.7275	0.1967
BIO16	0.1239	0.3835	-0.1683	-0.1296	0.1403	0.1392	0.1243	-0.1836	0.0655	-0.1829
BIO17	-0.1576	0.0342	-0.4124	0.0486	-0.3580	-0.1757	-0.1143	-0.0663	-0.1925	-0.0874
BIO18	0.2002	0.3205	-0.1778	-0.0530	0.0480	-0.2251	0.0636	-0.2422	0.1638	0.7614
BIO19	-0.2121	0.0520	-0.2608	-0.0952	-0.2542	0.5994	0.1452	0.5460	0.2139	0.2615
EL	0.0454	-0.0599	0.0407	-0.7652	-0.0704	-0.0342	-0.6153	0.0142	0.1178	-0.0021

Table S7. Principal component loadings table for *C. campestris*.

	PC1	PC2	PC3	PC4	PC5	PC6	PC7	PC8	PC9	PC10
BIO1	-0.3538	-0.1221	0.0635	-0.1556	0.0950	-0.0079	0.0893	0.0541	0.1909	0.1078
BIO2	-0.0729	-0.2893	0.2193	-0.0175	-0.2910	-0.4193	-0.2989	-0.3831	-0.0556	-0.0823
BIO3	-0.3054	-0.1214	-0.0876	0.1510	-0.0068	-0.3430	-0.3630	-0.2054	-0.0142	0.0450
BIO4	0.3053	-0.0081	0.3038	-0.1622	-0.1157	0.1540	0.0451	0.0721	0.0024	-0.0213
BIO5	-0.1743	-0.2433	0.2865	-0.3523	-0.1370	0.0053	0.1008	-0.0169	0.0043	0.1964
BIO6	-0.3531	-0.0307	-0.1922	-0.0567	0.1460	0.0221	0.0963	0.0644	0.0665	0.1306
BIO7	0.2509	-0.1158	0.3666	-0.1553	-0.2298	-0.0191	-0.0363	-0.0751	-0.0644	-0.0132
BIO8	-0.0605	0.0088	0.3679	-0.0614	0.5397	-0.1636	-0.3952	0.4838	-0.2983	-0.2167
BIO9	-0.2878	-0.1502	-0.1606	-0.1494	-0.2455	0.0982	0.2872	0.0388	-0.5117	-0.6384
BIO10	-0.2219	-0.1677	0.2981	-0.3509	0.0175	0.1172	0.1869	0.1314	0.1961	0.1393
BIO11	-0.3671	-0.0797	-0.1038	-0.0415	0.1050	-0.0631	0.0581	0.0134	0.0918	0.0877
BIO12	-0.1834	0.3702	0.1524	0.0087	-0.1272	-0.0043	0.0180	-0.0275	-0.1416	0.1293
BIO13	-0.2192	0.2555	0.2684	0.2045	-0.0712	0.1875	0.0551	-0.1284	-0.1853	0.0730
BIO14	-0.0037	0.3812	-0.0333	-0.2731	-0.0995	-0.2883	-0.0157	0.0549	0.3752	-0.2851
BIO15	-0.1608	-0.1828	0.2279	0.3862	-0.0759	0.3515	-0.1476	0.0476	0.5471	-0.4768
BIO16	-0.2214	0.2703	0.2500	0.2032	-0.0844	0.1653	0.0167	-0.1177	-0.1635	0.1133
BIO17	-0.0301	0.3840	-0.0340	-0.2869	-0.1166	-0.2622	0.0438	0.0667	0.1725	-0.2110

BIO18	-0.0936	0.3134	0.3059	0.1402	0.2098	-0.1511	0.2234	-0.2942	0.0365	-0.1073
BIO19	-0.1681	0.2146	-0.1184	-0.0949	-0.4758	0.2501	-0.5144	0.3266	-0.0358	0.1570
EL	-0.0043	-0.1005	0.1192	0.4540	-0.3301	-0.4520	0.3591	0.5530	0.0195	0.1325

Table S8. PCA variance explained for *C. chinensis*.

	PC1	PC2	PC3	PC4	PC5	PC6	PC7	PC8	PC9	PC10
Standard Deviation	2.9299	2.1680	1.6587	1.3806	0.9041	0.6489	0.5885	0.4065	0.3556	0.3205
Proportion of Variance	0.4292	0.2350	0.1376	0.0953	0.0409	0.0211	0.0173	0.0083	0.0063	0.0051
Cumulative Proportion	0.4292	0.6642	0.8018	0.8971	0.9380	0.9590	0.9763	0.9846	0.9909	0.9961

	PC11	PC12	PC13	PC14	PC15	PC16	PC17	PC18	PC19	PC20
Standard Deviation	0.1920	0.1299	0.1130	0.0703	0.0592	0.0464	0.0377	0.0177	0.0056	0.0002
Proportion of Variance	0.0018	0.0008	0.0006	0.0003	0.0002	0.0001	0.0001	0.0000	0.0000	0.0000
Cumulative Proportion	0.9979	0.9987	0.9994	0.9996	0.9998	0.9999	1	1	1	1

Table S9. PCA variance explained for *C. campestris*.

	PC1	PC2	PC3	PC4	PC5	PC6	PC7	PC8	PC9	PC10
Standard Deviation	2.5966	2.2067	1.7019	1.3537	1.1672	1.0937	0.6242	0.5319	0.3935	0.3451
Proportion of Variance	0.3371	0.2435	0.1448	0.0916	0.0681	0.0598	0.0195	0.0142	0.0077	0.0060
Cumulative Proportion	0.3371	0.5806	0.7254	0.8170	0.8852	0.9450	0.9644	0.9786	0.9863	0.9923

	PC11	PC12	PC13	PC14	PC15	PC16	PC17	PC18	PC19	PC20
Standard Deviation	0.2368	0.2039	0.1456	0.1234	0.1073	0.0664	0.0567	0.0316	0.0152	0.0005
Proportion of Variance	0.0028	0.0021	0.0011	0.0008	0.0006	0.0002	0.0002	0.0001	0.0000	0.0000
Cumulative Proportion	0.9951	0.9972	0.9982	0.9990	0.9996	0.9998	0.9999	1.0000	1.0000	1.0000

Table S10. PCA variance explained for *C. australis*.

	PC1	PC2	PC3	PC4	PC5	PC6	PC7	PC8	PC9	PC10
Standard Deviation	2.7486	2.1857	1.9442	1.1804	0.9884	0.7924	0.6033	0.4925	0.3323	0.2170
Proportion of Variance	0.3777	0.2389	0.1890	0.0697	0.0488	0.0314	0.0182	0.0121	0.0055	0.0024
Cumulative Proportion	0.3777	0.6166	0.8056	0.8753	0.9241	0.9555	0.9737	0.9858	0.9913	0.9937

	PC11	PC12	PC13	PC14	PC15	PC16	PC17	PC18	PC19	PC20
Standard Deviation	0.2035	0.1665	0.1589	0.1291	0.0949	0.0631	0.0418	0.0207	0.0075	0.0004
Proportion of Variance	0.0021	0.0014	0.0013	0.0008	0.0005	0.0002	0.0001	0.0000	0.0000	0.0000

Cumulative Proportion	0.9958	0.9971	0.9984	0.9992	0.9997	0.9999	1.0000	1.0000	1.0000	1.0000

Table S11. The width of three subg. *Grammica* species on each principal component axis in the PCA space.

	PC1	PC2	PC3	PC4	PC5	PC6	PC7	PC8	PC9	PC10
C. chinensis	13.812	10.404	8.172	10.759	6.106	4.777	3.297	4.425	3.151	2.743
C. campestris	22.097	25.515	10.208	13.575	10.780	8.427	7.665	5.421	4.375	4.087
C. australis	15.410	15.933	17.040	9.705	8.430	7.978	5.935	4.353	4.296	2.406

	PC11	PC12	PC13	PC14	PC15	PC16	PC17	PC18	PC19	PC20
C. chinensis	1.190	0.774	0.832	0.434	0.447	0.261	0.244	0.114	0.028	0.001
C. campestris	3.151	4.791	1.662	1.535	1.836	1.110	0.719	0.356	0.193	0.002
C. australis	2.073	1.489	1.259	0.952	0.945	0.532	0.329	0.181	0.071	0.002
